# The Relationship Between Multidimensional Motivation and Endocrine-Related Responses: A Systematic Review

**DOI:** 10.1177/1745691620958008

**Published:** 2021-01-29

**Authors:** Richard P. Steel, Nicolette C. Bishop, Ian M. Taylor

**Affiliations:** 1 Department of Psychology, Nottingham Trent University; 2 Department of Sport, Exercise, and Health Sciences, Loughborough University

**Keywords:** stress, HPA axis, hormone, immune functioning

## Abstract

Multidimensional motivational theories postulate that the type of motivation is as important as the quantity of motivation, with implications for human functioning and well-being. An extensive amount of research has explored how constructs contained within these theories relate to the activation of the endocrine system. However, research is fragmented across several theories, and determining the current state of the science is complicated. In line with contemporary trends for theoretical integration, this systematic review aims to evaluate the association between multidimensional motivational constructs and endocrine-related responses to determine which theories are commonly used and what inferences can be made. Forty-one studies were identified incorporating five distinct motivation theories and multiple endocrine-related responses. There was evidence across several theories that high-quality motivation attenuated the cortisol response in evaluative environments. There was also evidence that motivational needs for power and affiliation were associated with lower and higher levels of salivary immunoglobulin A, respectively. The need for power may play a role in increasing testosterone when winning a contest; however, this evidence was not conclusive. Overall, this review can shape the future integration of motivational theories by characterizing the nature of physiological responses to motivational processes and examining the implications for well-being.

Motivation can be defined as the process invoking the energization and direction of behavior toward positive stimuli (Elliot, 2006). Many traditional definitions of motivation adopted a unidimensional perspective in which the strength or magnitude of the energizing belief is the key determinant in driving behavior. In other words, a greater quantity of motivation yields a greater likelihood of initiating behavior and meeting goals (Elliot, 2006). In addition to these behavioral consequences, the magnitude of motivation has long been associated with physiological responses (e.g., Walter Cannon’s theories examining physiological responses to pain, hunger, fear, and rage; Cannon, 1929). Increased motivation for behavioral engagement has also been associated with elevated aspects of cardiovascular functioning (Wright & Gendolla, 2012). The investigation of motivational processes, therefore, helps us understand not only goal-oriented behavior but also the health and physical functioning of humans.

This simple emphasis on the quantity of motivation differs from multidimensional classifications that convey not only the magnitude of motivation but also the qualitatively different reasons that motivate people to engage in goal-directed behavior (McClelland, 1987; Ryan & Deci, 2017). To illustrate this point, one might observe a student reading a book in a university library and determine that he or she is motivated to read; however, one would not be able to determine whether they are reading for an upcoming assessment, for personal development, or for pleasure. For example, intrinsic motivation describes activities that individuals pursue for reasons that are inseparable from the activity itself, whereas extrinsic motivation refers to engagement to attain (or avoid) separable consequences or external contingencies (Ryan & Deci, 2017). These different types of motivation have implications for behavior, functioning, and well-being. Although both motivational dispositions predict performance, intrinsic motives better predict the quality of performance (e.g., engaging in complex tasks that require creativity or attention to detail), whereas extrinsic motivation better predicts the quantity of performance (e.g., less complex tasks evaluated by counting discrete units of output; Cerasoli, Nicklin, & Ford, 2014).

More broadly, differentiating types of motivation helps us better understand engagement across multiple contexts, including education, work, human development, exercise, and sports (e.g., Deci & Ryan, 2014; Elliot, Dweck, & Yeager, 2018; Roberts & Treasure, 2012; Ryan, 2012; Shah & Gardner, 2008). Significant quantities of low-quality motivation can drive individuals to achieve behavioral outcomes but is often accompanied by psychological dysregulation, such as increased stress, depressive symptoms, and clinical disorders (e.g., de Bruin, Bakker, & Oudejans, 2009; Emery, Heath, & Mills, 2016; Tuominen-Soini, Salmela-Aro, & Niemivirta, 2008). In sum, behavior can be driven by (a) high-quality motivational processes that simultaneously facilitate well-being or (b) low-quality motivation that drives behavior but may have deleterious consequences for well-being (e.g., Dweck, 2017; Elliot, Murayama, & Pekrun, 2011; Ryan & Deci, 2017).

Comparing different theoretical perspectives and constructs could lead to an “apples and oranges” phenomenon in which fundamental differences make comparisons difficult and of limited worth. However, significant movements toward unifying theories of motivation have been made, which provide a framework to help avoid futile comparisons. Dweck (2017) suggested that a unified approach is necessary to address societal problems in more integrated ways rather than isolated theories explaining isolated phenomena. Likewise, it has been proposed that it is timely to integrate diverse efforts to understand motivation into a unified overview of human motivation (Uusberg, Suri, Dweck, & Gross, 2019). For example, psychological need-based perspectives describing the importance of achievement and competence-related motives, as well as a need to affiliate with others, can be integrated (Schüler, Baumann, Chasiotis, Bender, & Baum, 2019). In addition to drawing conclusions concerning similar constructs, integrative viewpoints can suggest where different constructs may still be compatible. For example, recent work has begun to integrate constructs from different theories into holistic models of motivation (Chen, Elliot, & Sheldon, 2019; Sieber, Flückiger, Mata, Bernecker, & Job, 2019). Divergence across theories can also be highlighted, including the difference between need-driven processes and mental representations of active goals (Dweck, 2017), as well as the focus on explicit or implicit motives (Schüler et al., 2019). Theories can also be distinguished according to their emphasis on individual differences in the motivational strength of particular psychological needs (McClelland, 1987) versus the degree to which the psychological needs are satisfied (Ryan & Deci, 2017). Therefore, although there are theoretical differences between these approaches, sufficient similarity and compatibility between theories is apparent, and this has stimulated a scientific movement toward adopting a more systematic, integrated approach.

Although there are a variety of ways in which to explore the degree of similarity and compatibility between theories, one intriguing method is by examining physiological responses. A substantial amount of research in motivational science has investigated how psychological processes influence downstream physiological mechanisms implicated in motivating human behavior and improved health (i.e., how different types of motivation relate to physiological responses). This research has focused particularly on the activation of the endocrine system and the subsequent secretion of hormones that modulate human behavior and help maintain homeostasis. Motivational processes play a key role in regulating hormone secretion, especially in response to a stressful situational threat to goal-directed behavior (Carver & Scheier, 1999; Dickerson & Kemeny, 2004). Hormones are responsible for the regulation of many activities, including metabolism, immune functioning, reproductive processes, and circadian rhythms (Black, 1994; Tortora & Derrickson, 2016). The activation of the endocrine system has adaptive advantages; for example, when confronted with a stressor, activation of the hypothalamic-pituitary-adrenal (HPA) axis mobilizes the body’s resources to meet a challenge or threat (Lupien, McEwen, Gunnar, & Heim, 2009). However, the diversion of resources also has an immunosuppressive effect (Glaser & Kiecolt-Glaser, 2005; Segerstrom & Miller, 2004), which, if persistent, can lead to mental and physical health dysregulation and the pathogenesis of disease (McEwen & Stellar, 1993).

Despite the burgeoning volume of research in this area, a systematic review of the literature exploring multidimensional motivation and hormonal secretion remains absent. Research is fragmented across several motivational theories exploring a variety of hormonal responses, and therefore accurately assessing the current state of the literature is complicated. Researchers well versed in a particular theoretical perspective would benefit from a review highlighting complementary lines of investigation from similar or complementary theoretical perspectives to identify avenues for research. Hence, we aim to systematically review the research relating to theoretically derived motivational constructs and physiological responses observed in the endocrine system. Previous scientific approaches taken to study the psychophysiology of motivation have come under criticism. In many instances, unwarranted conclusions are made, and relationships between motivation and marker are overemphasized (Richter & Slade, 2017). Adopting a broad perspective to scrutinize the psychophysiological relationship between motivation and physiological response can illuminate the instances in which these problems may have occurred.

There are a variety of methods available for measuring endocrine-related responses, including the use of plasma, urine, and sweat. Although there are advantages and disadvantages to each of these methods of measurement, for social-science research, saliva offers more advantages than alternative methods. Salivary responses present a valid, reliable, and noninvasive method of reviewing acute and circadian patterns that limits the possibility of the method confounding the item of interest; for example, venipuncture can significantly increase cortisol levels (Smyth, Hucklebridge, Thorn, Evans, & Clow, 2013). Moreover, plasma collection requires specialist training, is time-consuming and expensive, and has ethical constraints (Kirschbaum & Hellhammer, 1994; Smyth et al., 2013). Thus, this article focuses on salivary markers of endocrine-related responses.^1^

## Method

The reporting of this systematic review adheres to the Preferred Reporting Items for Systematic Reviews and Meta-Analysis (PRISMA) statement (Moher, Liberati, Tetzlaff, Altman, & The PRISMA Group, 2009). The PRISMA statement aims to ensure consistency and transparent reporting of a systematic review and consists of 27 items to include when reporting a systematic review and a four-phase flow diagram detailing the process of identifying of studies included in the review (i.e., identification, screening, eligibility, inclusion).

### Search strategy

The databases used to search for relevant literature were Web of Science, PubMed, PsycINFO, and Scopus. Unpublished theses and dissertations were searched using ProQuest. The full-text and reference lists of extracted studies were also inspected for relevant literature. Search strategies were built around two groups of keywords: motivation terminology and endocrine-related responses. A scoping search was carried out before the formal screening process that uncovered potential research in the following theories: motive-disposition theory (MDT; McClelland, 1987); self-determination theory (SDT; Ryan & Deci, 2017), achievement goal theory (AGT; Nicholls, 1984), and implicit theory (Dweck, 2016). Keywords related to these theories were included in the search strategy. Motivational constructs were identified using the broad search term “motiv*,” the names of specific theories (e.g., “self-determination”), or associated keywords (e.g., “nPower”). Examples of keywords used to identify endocrine-related responses included general terms (e.g., “*endocrin*,” “hormon*”) and specific types of responses (e.g., “testosterone,” “cortisol”). A full list of search terms is included in the Supplemental Material available online.

### Inclusion criteria

Studies were required to (a) be published in the English language from 1970 up to and including May 2019; (b) have a quantitative measure of at least one salivary endocrine-related response taken from human participants of any age; and (c) contain a theoretically derived construct of multidimensional motivation either measured quantitatively using validated questionnaire data or an experimental manipulation of motivation. Unidimensional conceptualizations of motivation were excluded, such as effort as a motivational indicator within motivational intensity theory (Brehm & Self, 1989) and self-efficacy theory (Bandura, 1977),^2^ as were atheoretical measures of motivation (e.g., studies that used music as a motivational tool). No exclusion criteria were set in relation to participant characteristics or the study sample size.

### Identification of relevant studies

Citation abstracts and full-text articles, together with screening questions, were uploaded to Covidence, an Internet-based software program that facilitates systematic reviewing. Duplicates were automatically removed in the first instance by the software program and in the second instance by the authors during the title and abstract screening.

**Fig. 1.**
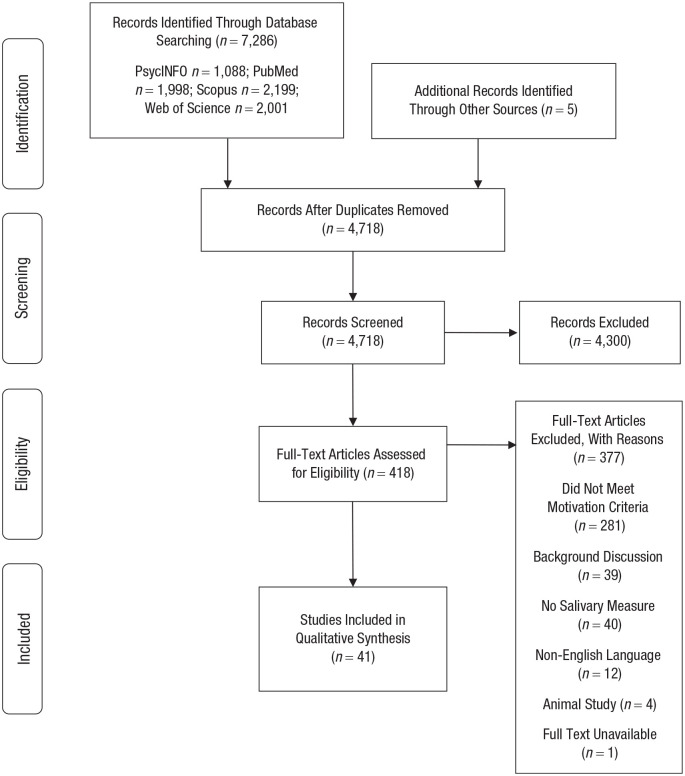
Preferred Reporting Items for Systematic Reviews and Meta-Analysis (PRISMA) flowchart information through the different phases of the systematic review.

Authors R. P. Steel and I. M. Taylor independently screened the results of the search strategy to determine whether the article met the inclusion criteria. All abstracts and titles were screened by these authors. If abstracts were not available or did not contain sufficient information, the full text was screened to determine potential eligibility. After the title and abstract screening were completed, the same authors subsequently examined the full text of potential studies to determine whether it met the final inclusion criteria. Disagreements over inclusion were resolved through discussion and adjudication by author N. C. Bishop; however, no disagreement occurred. None of the review authors was blind to the journal titles, study authors, or institutions. In accordance with the PRISMA statement, the different phases of the process are summarized in Figure 1.

### Data extraction and coding

To ensure consistency between R. P. Steel and I. M. Taylor, calibration exercises were conducted before starting the review. These two authors extracted data independently using standardized forms. R. P. Steel completed the data extraction for all included studies and I. M. Taylor randomly extracted data for 10 studies to confirm accuracy. Data extracted included the underpinning motivational theory, endocrine-related response, participant demographic information, study methodology, and sample size. Because of the broad range of motivational constructs and the hazards of pooling data from diverse, nonrandomized studies (Sterne, Egger, & Moher, 2011), a meta-analysis was deemed inappropriate for this review.

Observational and experimental studies were included in the review. A single measure of saliva indicated a cross-sectional study, with two or more collection time points treated as longitudinal data. Experimental studies included a manipulation of a motivation construct (e.g., the manipulation of autonomous regulation in line with SDT). If the experimental manipulation was not based on motivation but motivation was measured, the data extracted were classified as either cross-sectional or longitudinal. Primary experimental effects (e.g., analysis of variance statistics), longitudinal statistics inferring change (e.g., regression coefficients, controlling for baseline measures of the dependent variable), or correlational statistics (e.g., bivariate correlation) were extracted depending on the study design.

### Risk of bias/study quality

The risk of bias was assessed using a modified version of the Downs and Black (1998) checklist. The original checklist comprised 27 items measuring various aspects of quality assessment; however, 14 of these items were discarded because of a lack of relevance (items suited mainly to clinical trials). Of the remaining 13 items, three items were relevant only for experimental studies (e.g., blinding of participants). The scoring for each question followed the format of *yes* (1) and *no or unable to determine* (0); higher scores represented a low risk of bias (i.e., high quality).

## Results

### Study characteristics

The review included 40 published articles and one doctoral thesis for a total of 46 independent studies. Of these 46 studies, 29 were experimental, 11 were longitudinal, and six were cross-sectional designs. Twenty-nine studies used mixed-gender samples, with three studies not reporting gender. Studies that used samples from only men (*n* = 10) or only women (*n* = 4) did so to measure characteristics unique to that gender (e.g., medical contraceptive use, menstrual cycle) or physiological hormones that vary depending on gender (e.g., testosterone, progesterone, estradiol). In total, there were five motivational theories included in the extracted studies: MDT (McClelland, 1987; *n* = 30), SDT (Ryan & Deci, 2017; *n* = 5), AGT (Nicholls, 1984; *n* = 4), implicit theory (Dweck, 2016; *n* = 4), and reversal theory (Apter, 2001; *n* = 3). Nine salivary hormonal or endocrine-related responses were measured: cortisol (*n* = 26), testosterone (*n* = 11), salivary secretory immunoglobulin A (sIgA; *n* = 6), progesterone (*n* = 5), estradiol (*n* = 5), salivary α-amylase (sAA; *n* = 2), epinephrine (*n* = 2), dehydroepiandrosterone (DHEA; *n* = 2), and norepinephrine (*n* = 1). When assessing the risk of bias, most studies scored relatively high (i.e., low risk of bias), with experimental studies scoring an average of 10.23 (out of 13) and observational (i.e., cross-sectional and longitudinal) studies scoring 7.73 (out of 10). The risk of bias for individual elements from the Downs and Black (1998) checklist is summarized in the Supplemental Material. However, it is important to note that the blinding of researchers (four studies of 26), reporting of exact *p* values (24 studies of 41), and reporting of power analyses (five studies of 41), all of which are recommended practices, were relatively poorly observed across the reviewed articles.

### Primary results

A summary of all the key data extracted is presented in Table 1. The studies are grouped by theory (e.g., MDT), and data include the lead author, year of publication, study design, sample size, study features, quality score (risk of bias), endocrine response, motivational construct, statistical significance and direction of any relationship, and supplementary comments. The following sections provide a description of each underpinning theory together with a commentary on the findings.

**Table 1.** Summary of Key Study Characteristics of Reviewed Studies

Study	Study design	*N* (F)	Study features (mean age)	Quality score	Endocrine response (data points)	Motive measure^a^	Motivational construct (significance/direction)	Comments
Motive-disposition theory								
Dabbs et al. (1990)	CS	110 (53)	College students (n.r.)	6/10	T (1)	TAT	nPow (n.s.)	
Edelstein et al. (2010)	CS	102 (44)	Undergraduate students (18.79)	8/10	E (1)	PSE	nAff (n.s.)	
Jemmott et al. (1983)	Long.	64 (16)	Dental students (23.4); 10-month longitudinal	7/10	sIgA (5)	TAT	nAff (+), nPow (–)	The relationship between nAff and sIgA was higher than the relationship between nPow and sIgA across all time points.
McClelland et al. (1980)	Long.	27 (0)	College sophomores (n.r.)	6/10	sIgA (2)	PSE	nPow (n.r.), nAff (n.r.)	The direct relationships independent of stress were not reported.
McClelland et al. (1982)	CS	133 (0)	Male prisoners (28.5)	8/10	sIgA (1)	TAT	nPow (n.r.), nAff (n.r.)	The direct relationships independent of stress were not reported.
McClelland et al. (1985)	Long.	46 (17)	College students (n.r.); examination stress	7/10	sIgA, NE (3)	TAT	IgA: nPow (–), nAff (n.s.) NE: nPow (+), nAff (n.s.)	sIgA: nPow-dominant participants experienced a reduction in sIgA 105 min after the exam. This difference was significant compared with the baseline and compared with nAff-dominant participants. NE: Increased in nPow-dominant participants after the exam.
McClelland et al. (1987)	Exp.	61 (30)	University students (19.0); emotional-arousal film: nPow vs. nAff	6/13	C, EP, NE (2)	PSE	nPow (n.s.), nAff (n.s.)	
McClelland & Kirshnit (1988)	Exp.	132 (n.r.)	College students (n.r.); emotional-arousal film: nPow vs. nAff	9/13	sIgA (3)	TAT	nPow (n.s.), nAff (+)	
Oxford et al. (2017)	Exp.	326 (161)	University students (21); contest outcome: win vs. loss	11/13	C, E, P, T	PSE	T and nPow: win NC (+), loss male (+), loss NC (–) E and nPow: loss NC (–)	Post hoc results revealed that nPow predicted higher cortisol for men in losing teams and that there was a negative association between nPow and cortisol in individual women. In women only, nAff predicted a postcontest decline in progesterone in individual contests, and nAff weakly predicted increasing progesterone for team contests.
Schultheiss et al. (1999)	Exp.	42 (n.r.)	University students (20.26); contest outcome: win vs. loss	8/13	T (3)	PSE	pPow: win (+), loss (n.s.), p + sPow: win (–), loss (n.s.)	pPow refers to participants for whom sPow was absent; p + sPow refers to participants with high levels of pPow who also exhibited sPow.
Schultheiss & Rohde (2002)	Exp.	66 (0)	Vocational college students (23.83); contest outcome: win vs. loss	9/13	T (6)	PSE	nPow win (n.r.), loss (n.r.)	Direct effect of condition; nPow and testosterone were not reported; nPow predicted a significant increase in testosterone from Times 3 to 5 among winners with low levels of inhibition.
Schultheiss et al. (2003)	Long.	54 (18)	University students (n.r.)	7/10	T, P, E (3)	PSE	nAff (n.r.), nPow (n.r.)	The study examined contextual effects related to menstrual-cycle and relationship status. Direct relationships independent of these variables were not reported.
Schultheiss et al. (2004)	Exp.	60 (39)	Undergraduate students (19.78); emotional-arousal film: nAff vs. nPow vs. control	10/13	P, T (3)	PSE	nAff (n.s.), nPow (n.s.)	Main effect for experimental condition not significant. Post hoc analysis revealed that the nAff condition experienced higher postfilm progesterone levels than the neutral condition. Further post hoc analysis of Time 5 found that baseline testosterone predicted decreased postfilm testosterone in the nAff condition, increased testosterone in the nPow condition in males, and decreased nPow testosterone for women.
Schultheiss et al. (2005, Study 1)	Exp.	95 (0)	Undergraduate students (19.67); contest outcome: win vs. loss	10/13	T (6)	PSE	nPow win (n.s.), nPow loss (n.s.)	Main effect for experimental condition was not significant. Post hoc analysis revealed a negative correlation between nPow and testosterone in the losing condition and a positive nPow/testosterone trend in the losing condition.
Schultheiss et al. (2005, Study 2)	Exp.	75 (75)	Undergraduate students (20.82); contest outcome: win vs. loss	—	T (6)	PSE	nPow win (n.s.), nPow loss (n.s.)	Main effect for experimental condition was not significant. Post hoc analysis revealed a positive nPow/testosterone association in the losing condition at Time 4.
Schultheiss et al. (2012)	CS	92 (50)	University students (23)	9/10	C, P (1)	PSE/PGI	nPow (n.r.), nAff (n.r.), nAch (n.r.)	Direct associations were not reported—only the difference between explicit and implicit motivation.
Schultheiss et al. (2014, Study 1)	Long.	108 (53)	University students (20)	10/13	C (2)	PSE	nAch (–)	Reanalysis of Wirth, Welsh, & Schultheiss (2006) results, excluding win/loss manipulation.
Schultheiss et al. (2014, Study 2)	Exp.	62 (31)	University students (23.87); TSST vs. control	—	C (2)	PSE	nAch (–)	nAch predicted a reduced cortisol response to TSST that was absent in the control task.
Slatcher et al. (2011)	CS	74 (0)	Psychology undergraduates (n.r.)	9/10	T (1)	See note	nPow (n.s.)	nPow was determined by four judges who assessed dominance behavior via videotape.
Stanton & Schultheiss (2007)	Exp.	49 (49)	University students (19.96); contest outcome: win vs. loss	10/13	T, E (6)	PSE	T: nPow win (n.s.), loss (n.s.); E: nPow win (n.s.), loss (n.s.)	Data drawn from Wirth et al. (2006, Study 2). A post hoc positive association between nPow and estradiol was observed in the winning condition when aggregating Times 4–6.
Stanton & Edelstein (2009)	Long.^b^	40 (40)	University students (18.58)	11/13	E (2)	PSE	nPow (+)	
Vongas & Al Hajj (2017, Study 1)	Exp.	84 (0)	Undergraduate students (22.01); contest outcome: win vs. loss	11/13	T (3)	PSE	pPow win (–), loss (n.s.)	pPow moderated the relationship between competition outcome and testosterone change. Increased pPow in the winning condition attenuated the decrease in testosterone.
Vongas & Al Hajj (2017, Study 2)	Exp.	72 (0)	Undergraduate students (21.39); contest outcome: win vs. loss	—	T (3)	PSE	pPow win (–), loss (n.s.)	pPow moderated the relationship between competition outcome and testosterone change. Increased pPow in the winning condition attenuated the decrease in testosterone.
Wegner et al. (2014)	Exp.	59 (32)	High school students (14.8); task: physical vs. psychosocial vs. control	11/13	C (2)	OMT	nAff: psychosocial (–), physical (n.s.), control (n.s.)	The psychosocial task is the most appropriate measure as it is unlikely the physical task (15 mins moderate running) or control would be sufficient to provoke a cortisol response.
Wegner et al. (2015)	Exp.	57 (33)	High school students (14.8); task: physical vs. psychosocial vs. control	11/13	C (2)	OMT	nPow: psychosocial (+), physical (n.s.), control (–)	The psychosocial task is the most appropriate measure because it is unlikely that the physical task (15 min of moderate running) or control would be sufficient to provoke a cortisol response.
Wiemers et al. (2015)	Exp.	72 (34)	University sample (n.r.); TSST vs. control	10/13	C, sAA (4)	PSE	C × nPow (+), C × nAch (n.s.), C × nAff (n.s.), sAA × nPow (+), sAA × nAch (n.s.), sAA × nAff (n.s.)	
Wirth & Schultheiss (2006)	Exp.	87 (38)	Undergraduate sample (19.7); emotional-arousal film: HoC vs. FoR vs. control	10/13	C, P (3)	PSE	C × nPow (n.s.), C × nAff (+), C × nAch (n.s.), P × nPow (n.s.), P × nAff (+), P × nAch (n.s.)	nAff positively predicted postfilm progesterone in the FoR condition.
Wirth et al. (2006, Study 1)	Exp.	66 (0)	Vocational college (23.8); contest outcome: win vs. loss	9/13	C (6)	PSE	nPow win (n.s.), loss (+)	
Wirth et al. (2006, Study 2)	Exp.	108 (53)	University students (20.3); contest outcome: win vs. loss	—	C (6)	PSE	nPow win (n.s.), loss (n.s.)	Post hoc analysis aggregated pretest (Times 1–3) and posttest (Times 4–6) cortisol. nPow positively predicted cortisol response in males in the losing condition but not in winning condition. Post hoc analysis revealed evidence of an effect in participants tested after 2 p.m.
Yang et al. (2015)	Exp.	50 (26)	University students (M = 19.9, F = 18.8); D2 test of attention: positive vs. negative vs. no feedback	11/13	C (2)	PSE	nAch (n.s.)	Study was experimental; however, the manipulation was not related to motivation. Residualized cortisol was significantly associated with the positive feedback condition.
Self-determination theory								
Bartholomew, Ntoumanis, Ryan, Bosch, & Thøgersen-Ntoumani (2011)	CS	120 (92)	Junior athletes (14.51)	8/10	sIgA (1)	See note	Need satisfaction (n.s.), need thwarting (+)	Need satisfaction assessed using a composite scale comprising IMI, NfRS, and NfA. Need thwarting assessed using PNTS.
Quested et al. (2011)	Long.	61 (41)	Ballet dancers (19.3); challenge vs. threat appraisal	9/10	C (5)	See note	Need satisfaction (–)	Need satisfaction was not significant when challenge appraisals were included in the analysis. Need satisfaction assessed using a composite scale comprising IMI, NfRS, and NfA.
Reeve & Tseng (2011)	Exp.	78 (53)	Undergraduates (n.r.); puzzle solving: autonomous vs. controlled vs. neutral	9/13	C (3)	LCQ	Autonomy-supportive (–), controlled motivation (+), neutral motivation (n.s.)	
Sieber et al. (2016)	Exp.	69 (34)	Middle school children (14.16)	11/13	sAA (3)	PSE	Autonomy-supportive (–), autonomy-restrictive (+), control group (+)	Study integrated SDT (need satisfaction) and MDT (need strength). Experimental manipulation check was not conducted.
Sørensen et al. (2015)	Exp.	97 (85)	Health-care professionals (M = 44.5, F = 42.5)	13/13	C (4)	IMI	Intrinsic motivation (n.r.)	The study measured both cortisol and the IMI; however, the two measures were dependent variables. The relationship between the two was not an aim of the study and was not reported.
Achievement goal theory								
Breske et al. (2017)	Exp.	38 (0)	University students (20.68); ego-oriented environment	10/13	C (5)	PMCEQ	Control group (+), task-orientation intervention (–)	Significant Intervention × Time interaction. Cortisol response increased in the control condition compared with the task-orientation intervention.
Hogue et al. (2013)	Exp.	107 (61)	University students (19.89); juggling task: task vs. ego	10/13	C (7)	PMCSQ	Ego orientation (+), task orientation (–)	Significant Climate × Time interaction. Cortisol response increased in the ego condition compared with the task condition.
Hogue et al. (2017)	Exp.	47 (26)	Middle school students (11.98); juggling task: task vs. ego	11/13	C (4)	PMCSQ	Ego orientation (+), task orientation (–)	Significant Climate × Time interaction. Cortisol response increased in the ego condition compared with the task condition.
Rozek (2014)	Exp.	78 (34)	University students (n.r.)	8/13	C (4)	Not reported	Mastery environment (n.s.), performance environment (n.s.)	Experimental manipulation involved participants reading instructions that emphasized either mastery or performance goals to create the environment.
Implicit theory								
Calvete et al. (2019)	Exp.	503 (243)	Middle school students (14.53)	13/13	C (3), DHEA (3)	ETPB	Incremental beliefs (n.s.)	Overall intervention effect was absent; however, a significant attenuated DHEA response was observed between conditions for middle school children in Grade 8.
Lee et al. (2019)	Long.	499 (272)	High school students (14.2)	10/10	C (11)	ToIS	Incremental beliefs (–), entity beliefs (+)	Entity theory of intelligence predicted increased cortisol when grades declined. Incremental theory predicted lower cortisol across days and lower cortisol after an intense academic stressor.
Yeager et al. (2016, Study 1)	Exp.	60 (27)	High school students (15.61); learning task: incremental vs. control	13/13	C (2)	Not measured	Incremental beliefs (–)	Cortisol declined significantly in the implicit theory incremental intervention compared with the control group. Motive measure (manipulation check) was not conducted.
Yeager et al. (2016, Study 3)	Exp.	205 (n.r.)	High school students (n.r.); learning task: incremental vs. control	—	C, DHEA (6)	Not measured	Incremental beliefs (n.s.)	Overall, the intervention effect was absent; however a significant attenuated cortisol and DHEA response was observed between conditions on days 8 and 9. Motive measure (manipulation check) was not conducted.
Reversal theory								
Cuevas et al. (2014)	Long.	94 (94)	Breast cancer survivors (56.2)	8/10	C (10)	AMSP	Telic/paratelic (n.s.), conformist/negativistic (n.s.), mastery/sympathy (n.s.), autic/alloic (n.s.)	Participants displayed telic, conformist, sympathetic, and alloic dominance.
Filaire et al. (2007)	Long.	10 (0)	Elite paragliders (27.2)	7/10	C (6)	TDS	Serious-mindedness (n.s.), planning orientation (n.s.), arousal avoidance (n.s.)	The only significant finding across six times was association with serious-mindedness at Time 4 (*r* = −0.73).
Thatcher et al. (2003)	Long.	23 (1)	Skydivers (n.r.)	7/10	C (2)	AMSP	Telic/paratelic (n.s.), negativistic/conformist (n.s.), arousal seeking/arousal avoidance (n.s.)	Participants displayed conformist and arousal-seeking dominance. They were neither telic- or paratelic-dominant.

Note: AMPS = Apter Motivational Style Profile (Apter et al., 1998); C = cortisol; CS = cross-sectional; DHEA = dehydroepiandrosterone; E = estradiol; EP = epinephrine; ETPB = entity theory of personality beliefs (Yeager, Trzesniewski, Tirri, Nokelainen, & Dweck, 2011); Exp. = experimental; F = females; FoR = fear of rejection; HoC = hope of closeness; IMI = Intrinsic Motivation Inventory (Deci, Eghrari, Patrick, & Leone, 1994); LCQ = Learning Climate Questionnaire (Williams, Grow, Freedman, Ryan, & Deci, 1996); Long. = longitudinal; M = males; MDT, motive-disposition theory; nAch = need for achievement; nAff = need for affiliation; NC = normally cycling women; NE = norepinephrine; NfA = need for autonomy (Deci et al., 2001); NfRS = Need for Relatedness Scale (Richer & Vallerand, 1998); nPow = need for power; n.r. = not reported; OMT = Operant Multimotive Test (Kuhl & Scheffer, 1999); P = progesterone; PGI = Personal Goals Inventory (Brunstein, Schultheiss, & Grässman, 1998); PMCEQ = Perceived Motivational Climate in Exercise Questionnaire (Moore, Brown, & Fry, 2015); PMCSQ = Perceived Motivational Climate in Sport Questionnaire (Selfriz, Duda, & Chi, 1992); PNTS = Psychological Need Thwarting Scale (Bartholomew, Ntoumanis, Ryan, Bosch, & Thøgersen-Ntoumani, 2011); pPow = personal need for power; PSE = picture-story exercise (Schultheiss, Liening, & Schad, 2008); sAA = salivary α-amylase; SDT, self-determination theory; sIgA = salivary secretory immunoglobulin A; sPow = social need for power; T = testosterone; TAT = Thematic Apperception Test (Murray, 1943); TDS = Telic Dominance Scale (Morgatroyd, Rushton, Apter, & Ray, 1978); ToIS = Theory of Intelligence Scale (Dweck, 2016); TSST = Trier Social Stress Test; – = negative association; + = positive association.

a For experimental studies, this represents the manipulation check.

b Stanton and Edelstein (2009) took saliva measures at two time points; however, they were within an hour or one another, and the two measures were collapsed for analysis with a correlation reported between estradiol and nPow.

#### MDT

MDT draws heavily from Freud’s work; therefore, motivation is typically conceptualized as unconscious, and implicit measures are adopted. This approach differs considerably from other theories in this review. The three fundamental motives that energize and direct behavior within MDT are the need for power (nPower), the need for achievement (nAchievement), and the need for affiliation (nAffiliation). nPower is the motivation to exert influence over others. The use of power may be manipulative and controlling but may also be used to help and support others (Busch, 2018). nAchievement describes the motivation to reach a standard of excellence while avoiding goals that are excessively difficult or too easily achieved (Brunstein & Heckhausen, 2018). Finally, nAffiliation is the desire to establish and maintain close bonds and a sense of belonging through individual relationships and social connectedness (Hofer & Hagemeyer, 2018). These needs are considered fundamental to all humans but are assumed to differ in strength between cultures and individuals (McClelland, 1987).

Thirty studies used methods designed to measure or manipulate the needs for achievement, power, or affiliation. Most of the studies included in the review focused on one or two of these needs. Needs were generally assessed using the picture-story exercise (Schultheiss, Liening, & Schad, 2008) or the Thematic Apperception Test (Murray, 1943). These procedures involve presenting a series of pictures to participants, who then write a story for each. The participant’s stories are then coded for implicit motives using standardized instructions (Smith, 1992). Of the six cross-sectional studies, one study demonstrated a significant positive association between nPower and estradiol (Stanton & Edelstein, 2009). Three studies reported nonsignificant associations between nPower and cortisol (Dabbs, Hopper, & Jurkovic, 1990), nPower and testosterone (Slatcher, Mehta, & Josephs, 2011), and nAffiliation and estradiol (Edelstein, Stanton, Henderson, & Sanders, 2010), respectively. Two studies did not report the associations between implicit needs and sIgA (McClelland, Alexander, & Marks, 1982), cortisol, or progesterone (Schultheiss, Patalakh, & Rösch, 2012). However, for Schultheiss and colleagues (2012), incongruence between implicit and explicit needs (measured using the Personal Goals Inventory; Brunstein, Schultheiss, & Grässman, 1998) was positively associated with progesterone, but no relationship was observed between motivational incongruence and cortisol.

Most of the experimental and longitudinal studies that investigated MDT were undertaken by the same group of researchers and measured multiple motivational constructs and endocrine-related responses. The program of research began with evidence indicating that seven college students who had a high nPower, inhibition, and power stress profile exhibited lower levels of sIgA relative to a comparison group of 19 participants; however, the isolated relationship between nPower and sIgA was not reported (McClelland, Floor, Davidson, & Saron, 1980). A subsequent longitudinal study revealed that dental students who were nPower-dominant (high in nPower, low in nAffiliation) experienced significantly lower sIgA levels over a 10-month period compared with students who were nAffiliation-dominant (high in nAffiliation, low in nPower), especially during the summer examination period (Jemmott et al., 1983). In a further longitudinal study undertaken during an exam, students who were nPower-dominant (vs. nAffiliation-dominant) experienced significantly reduced levels of sIgA 105 min after the exam. However, there was no significant difference immediately after the exam or differences in epinephrine between groups (McClelland, Ross, & Patel, 1985). Finally, Schultheiss, Dargel, and Rohde (2003) observed nPower and nAffiliation along with testosterone, progesterone, and estradiol at three time points. Direct effects independent of relationship status and gender were not reported. However, when averaged across the time points, nPower was positively correlated with testosterone in men and single women and positively correlated with estradiol in engaged women, and nAffiliation was negatively correlated with progesterone in men.

Further research experimentally explored MDT by using excerpts of films designed to arouse implicit needs. Examples of films used include *The Bridges of Madison County* (Eastwood, 1995; arousal of nAffiliation) and *The Godfather II* (Coppola, 1974; arousal of nPower). The first study revealed no associations among nPower and nAffiliation and norepinephrine, epinephrine, and cortisol (McClelland, Patel, Stier, & Brown, 1987, Study 1). In a subsequent study, a film presented to arouse nAffiliation led to a corresponding increase in sIgA after the film; however, a film presented to arouse nPower had no effect on sIgA (McClelland & Kirshnit, 1988). In a third study, films arousing nPower or nAffiliation had no effect on testosterone or progesterone (Schultheiss, Wirth, & Stanton, 2004) despite a post hoc analysis suggesting nuanced relationships. A final study used two films to experimentally arouse either a hope of closeness (approach affiliation) or fear of rejection (avoidance affiliation; Wirth & Schultheiss, 2006). nPower, nAffiliation, and nAchievement were measured before and after the task, with cortisol and progesterone the outcome of interest. Nonsignificant correlations were reported between nPower and nAchievement and endocrine-related responses in women and any motive and endocrine-related response in men. A post hoc analysis of the sample of females revealed baseline nAffiliation predicted postfilm increases in progesterone in the fear-of-rejection condition. Furthermore, there were positive relationships between baseline nAffiliation and cortisol and postfilm nAffiliation and progesterone in women across all time points irrespective of experimental conditions.

Several studies have considered the relationship between motivational implicit needs and endocrine response during a variety of social-evaluative tasks. A reanalysis of data from an earlier study (Wirth, Welsh, & Schultheiss, 2006) demonstrated that higher nAchievement predicted lower cortisol response during a visuomotor competition irrespective of the competitive outcome (Schultheiss, Wiemers, & Wolf, 2014, Study 1). This association was successfully replicated in response to the Trier Social Stress Test (TSST) relative to a control condition (Schultheiss et al., 2014, Study 2). A third study found that TSST-induced nPower predicted an increase in sAA and a relatively lower increase in cortisol relative to a friendly version of the TSST that did not induce nPower (Wiemers, Schultheiss, & Wolf, 2015). Two further social-evaluative tasks examined nAffiliation and nPower in relation to cortisol change in high school students across three experimental conditions: physical stress, psychosocial stress, and a control task. In the first study, higher nAffiliation negatively predicted cortisol change, and this was largely attributable to participants in the psychosocial-stress condition rather than the physical-stress and control conditions (Wegner, Schüler, & Budde, 2014). The same experimental design was used in a follow-up study examining nPower and cortisol (Wegner, Schüler, Schulz Scheuermann, Machado, & Budde, 2015). nPower was associated with a greater cortisol response in the psychosocial-stress group, a lower cortisol response in the control condition, and no observed change in the physical-stress condition. In a challenging social-evaluative cognitive task with positive, negative, or neutral bogus feedback, nAchievement did not predict cortisol response overall; however, nAchievement dampened the cortisol response in the negative-feedback condition (Yang, Ramsay, Schultheiss & Pang, 2015).

Seven studies examined individual differences in nPower and endocrine responses in the context of winning or losing a contest, often examining males and females separately because of relative hormonal differences. The experiment pits two participants in an artificial competition against one another on a cognitive task, with task difficulty manipulated so the predesignated winner has a significant advantage. The first study compared a relatively personalized need for power (pPower) to a relatively altruistic, socialized need for power (sPower; Schultheiss, Campbell, & McClelland, 1999). Whether winning a task or simply imagining winning, participants high in pPower but for whom sPower was absent experienced a significant increase in testosterone. Participants high in pPower and for whom sPower was present demonstrated no change in testosterone after imagining success and a significant negative testosterone response to winning the task. No association between either type of need for power or testosterone was observed in participants who lost the task.

Two further studies demonstrated very few significant relationships, and those that were reported were nuanced. In the first study, which used an all-male sample, nPower predicted increased testosterone among winners who were low in activity inhibition (i.e., the frequency of the use of the word “not” in the picture-story exercise, a tool primarily used to assess implicit needs) at the fifth of six measurement points (Schultheiss & Rohde, 2002). In a second study, males and females were tested separately. In the sample of males, a significant negative correlation between nPower and testosterone among losers, and a marginally significant positive correlation among winners, was observed at the fifth of six time points (Schultheiss et al., 2005, Study 1). In the sample of females, a significant positive nPower and testosterone association among losers at the fourth of six time points was observed, with no significant association observed among winners (Schultheiss et al., 2005, Study 2). Most recently, Vongas and Al Hajj (2017) conducted two studies using a sample of males in which they collected testosterone at three time points. Across both studies, winners’ testosterone levels decreased significantly; however, higher pPower among winners resulted in a relatively smaller decrease in testosterone compared with low pPower. No significant effects between pPower and testosterone were observed among losers.

Two further win/loss studies examined cortisol and estradiol as endocrine responses. In an all-male sample, nPower positively predicted cortisol response among losers but not among winners (Wirth et al., 2006, Study 1). In a mixed-gender sample, only a negative trend was observed between nPower and cortisol among winners tested after 2:00 p.m. but not losers (Wirth et al., 2006, Study 2). The mixed-gender sample from Wirth et al. (2006) was further analyzed by extracting estradiol in the female participants only (Stanton & Schultheiss, 2007). nPower had a positive association with estradiol among winners but not losers.

Most recently, a statistically high-powered study examined nPower and nAffiliation in the win/loss context with time, sex differences, and individual versus team competition also explored in relation to testosterone, estrogen, progesterone, and cortisol (Oxford, Tiedtke, Ossmann, Özbe, & Schultheiss, 2017). For males high in nPower, there were significant increases in testosterone when losing. Furthermore, in females who were not taking oral contraceptives, there was a trend for nPower predicting increased testosterone among winners, with a corresponding decline among losers in both testosterone and estradiol. There were nuanced findings when investigating individual and team conditions on cortisol, with nPower predicting higher cortisol for men in losing teams and a negative association between nPower and cortisol in individual women.

When examining nAffiliation, the main analysis revealed nonsignificant results in relation to testosterone, estradiol, and cortisol. However, when examining only female participants, nAffiliation predicted a postcontest decline in progesterone for women who competed individually, and nAffiliation weakly predicted progesterone increasing for women competing in teams.

#### Self-determination theory

According to SDT, humans are assumed to flourish to the extent that three basic psychological needs are satisfied: competence, autonomy, and relatedness (Ryan, 1995). Competence relates to feelings of personal mastery and operating effectively within a particular environment (Ryan & Deci, 2017). Relatedness concerns the feeling of connectedness, particularly the importance of integration and significance within social groups (Deci & Ryan, 2014). Autonomy represents behaviors that are self-endorsed and are congruent with one’s interests and values (Ryan & Deci, 2006). When satisfied, these needs facilitate autonomous motivation, well-being, and self-determined functioning (Ryan & Deci, 2000). On the other hand, when they are unsatisfied or actively thwarted, ill-being and controlled suboptimal motivation and functioning occur (Ryan & Deci, 2017).

One cross-sectional study, one longitudinal study, and three experimental studies used SDT as a framework to examine motivational constructs and endocrine-related responses. The cross-sectional study included satisfaction and thwarting of basic psychological needs, as well as coaching context variables, in a population of 120 junior athletes, with sIgA the physiological response of interest. Basic psychological need satisfaction and thwarting were assessed collectively; hence, the individual relationships with sIgA were not reported. Basic psychological need thwarting was positively associated with higher levels of sIgA, whereas psychological need satisfaction was unrelated (Bartholomew, Ntoumanis, Ryan, Bosch, & Thøgersen-Ntoumani, 2011). In the longitudinal study, higher basic psychological need satisfaction (again assessed collectively) was associated with lower cortisol measures before, during, and after the performance of a ballet routine in a population of dancers (Quested et al., 2011).

Two of the three experimental studies manipulated motivation as the independent variable. During a puzzle-solving task, a decline in cortisol in a condition supporting autonomous motivational regulation, an increase in cortisol in a condition fostering controlling motivational regulation, and no change in a neutral condition was observed (Reeve & Tseng, 2011). A further study investigated the moderating effect of implicit autonomous disposition on the sAA response to different motivation-inducing environments using similar methods commonly used in MDT research (i.e., the picture-story exercise; Sieber, Schüler, & Wegner, 2016). In an environment in which motivation was controlled, an increase in sAA was observed among participants who displayed a high autonomous disposition, and a decreased sAA response was observed in participants with a low autonomy disposition. This pattern was also observed in the control group. Conversely, in the autonomy-supportive condition, participants with a high autonomous disposition experienced a lower sAA response, whereas participants with a lower autonomy disposition experienced a higher sAA response. A third experimental intervention involved obstetric anesthesia training and was not related to motivation (Sørensen et al., 2015). Intrinsic motivation and cortisol were measured as dependent variables; however, the direct relationship between the two was not reported.

#### AGT

AGT is concerned with conceptions of ability in achievement contexts, which can be demonstrated in two ways. A mastery or task-oriented goal focus refers to framing ability relative to one’s own past performance and knowledge (i.e., the degree of improvement; Nicholls, 1984). In these instances, personal development and high effort are encouraged, mistakes are viewed as part of the learning process, and cooperation with others is seen as facilitative. On the other hand, a performance or ego-oriented goal focus refers to comparisons to a normative standard (Nicholls, 1984). Although these goal constructs have been expanded in recent years (e.g., Elliot et al., 2011), only the mastery versus performance distinction has been used in psychophysiological research.

Four studies measured cortisol concurrently with AGT-based constructs. Three of the studies were conducted by the same lab; of these three studies, two used nearly identical methods: one with university students (Hogue, Fry, Fry, & Pressman, 2013) and one with middle school students (Hogue, Fry, & Fry, 2017). The experimental method involved teaching the participants to juggle over the course of 30 min, with the research assistants emphasizing either a task-oriented or ego-oriented environment. The results of both studies revealed a significant Time × Environment interaction, with the ego-oriented environment inducing significantly higher cortisol levels 15 and 30 min after the intervention relative to the task-oriented environment. These methods and results were conceptually replicated, as participants who received an educational intervention on achievement goals experienced lower cortisol levels compared with a control group despite both groups being exposed to an ego-oriented climate (Breske, Fry, Fry, & Hogue, 2017). Finally, an unpublished doctoral dissertation experimentally manipulated achievement goals during a socially evaluative task (Rozek, 2014). The manipulation involved participants reading a script that emphasized either performance or mastery-related goals. Structural equation modeling revealed that the type of goal (mastery vs. performance) did not have a significant effect on the cortisol intercept of the cortisol slope. It should be noted that the experimental manipulation in this study was weak and not supported by a manipulation check.

#### Implicit theories

Like AGT, implicit theory focuses on achievement motivation (Dweck, 2016). Nonetheless, it differs from AGT by focusing on beliefs about the malleability of intelligence and other psychological phenomena. Individuals who hold an incremental or “growth” mindset consider intelligence to be malleable and are motivated to increase competence. They are also more likely to seek challenges and have higher persistence in the face of adversity (Dweck & Leggett, 1988). This self-referenced motivational process with a focus on persistence and personal development shares a close conceptual foundation to the AGT construct of mastery-oriented goals. Conversely, individuals who hold an entity or “fixed” mindset view intelligence as static and are inclined to seek positive judgment from others. They are also less likely to engage in, and will have lower persistence during, challenging tasks if their confidence in success is low (Elliott & Dweck, 1988).

Three research articles comprised four studies; three experimental studies and one observational study were grounded in implicit theories (Dweck, 2016). The studies examined implicit theories in relation to cortisol and DHEA in populations of high school students. In the first study, Yeager, Lee, and Jamieson (2016, Study 1) conducted a laboratory-based experiment with participants randomly assigned to either an incremental theory or active-control reading-and-writing exercise, followed by the Trier Social Stress Test (TSST; Kirschbaum, Pirke, & Hellhammer, 1993). The incremental group experienced a reduction in cortisol after the task, and the control group experienced an increase in cortisol. Yeager and colleagues’ follow-up study (Study 2) was a preregistered double-blind intervention using the same experimental protocol implemented among a larger pool of participants with saliva collected over a 9-day period. Although an overall intervention effect was absent, an attenuated cortisol and DHEA response in the incremental-belief condition (compared with the control condition) was observed on the final two days (days 8 and 9) of the intervention.

A similar double-blind randomized control trial conducted among high school adolescents in Grades 8 through 10 over a 12-month period failed to find an overall incremental-theory intervention effect on cortisol and DHEA compared with a control group (Calvete et al., 2019). However, a post hoc analysis found support for the study hypothesis in 8th-grade adolescents who displayed a lower increase in DHEA in the incremental-theory intervention compared with the control group. Finally, an observational study found a significant interaction between declining grades, implicit theories of intelligence, and cortisol (Lee, Jamieson, Miu, Josephs & Yeager, 2019). Specifically, students who held an entity theory of intelligence experienced an increase in cortisol levels when grades declined. Furthermore, an incremental theory of intelligence lowered cortisol levels the day after an intense academic stressor and was also associated with lower cortisol levels over time.

#### Reversal theory

Three longitudinal studies investigated reversal theory (Apter, 2001), with cortisol the outcome of interest. Reversal theory posits that individuals are dynamically motivated between mutually exclusive motivational states. An individual’s state can be telic (achievement) or paratelic (enjoyment), conformist (compliance) or negativistic (rebelliousness), arousal-seeking (excitement) or arousal avoidance (tranquility), autic mastery (personal power) or autic sympathy (personal affection), and alloic mastery (vicarious power) or alloic sympathy (vicarious affection). Motivation is typically measured using the Apter Motivational Styles Profile (Apter, Mallows, & Williams, 1998), and metamotivational dominance for each motivational state is calculated by subtracting one score from the other. Two studies revealed no significant associations between metamotivational dominance and cortisol in skydivers (Thatcher, Reeves, Dorling, & Palmer, 2003) or breast-cancer survivors (Cuevas et al., 2014). A third study reported one significant negative association between the telic subscale of serious-mindedness and cortisol 10 min before a paragliding flight (Filaire, Rouveix, Alix, & Le Scanff, 2007).

## Discussion

The aim of this systematic review was to investigate multidimensional motivational theories and constructs and their associations with salivary endocrine-related responses. Comparing across theoretical boundaries facilitates understanding. It is possible to explore whether the psychophysiological processes are congruent with the broad tenets of the respective theory and whether there are cross-theory trends regarding each endocrine-related response. In this section, we discuss the trends in the results of this systematic review as they apply to specific endocrine-related responses. Drawing on key theoretical research articles, we also discuss the key similarities, compatibility, and differences between the main theories to emerge from this review. The final goal of this review was to evaluate the quality of the research and to consider the methodological validity and conclusions made (see Richter & Slade, 2017).

Cortisol was the most frequently studied hormonal response, and changes as a function of the quality of motivation induced were consistently observed. Mastery-oriented goal involvement, support for autonomous motivational regulation, and incremental implicit theories are hypothesized to be high-quality motivational bases within their theoretical umbrella (AGT, SDT, and implicit theories, respectively). In experimental studies, these high-quality motives attenuated cortisol secretion in social-evaluative learning tasks compared with low-quality motivational foundations (Breske et al., 2017; Calvete et al., 2019; Hogue et al., 2013, 2017; Reeve & Tseng, 2011; Yeager et al., 2016). In most of these studies, the effect sizes were large, the risk of bias was relatively low, and the broad findings were supported in a preregistered study with excellent methodological rigor (Yeager et al., 2016, Study 2). Observational evidence regarding incremental implicit theories (Lee et al., 2019) and basic need satisfaction (Quested et al., 2011) also aligned with this idea. The qualitative nature of motives and the subsequent physiological impact identified in this review complement other biological links to multidimensional theories of motivation (e.g., Di Domenico & Ryan, 2017). This line of inquiry suggests that biological processes associated with improved human functioning that are integrated with motivational and psychological theory can enhance the validity and improve understanding of the mechanisms underpinning these processes (Ryan & Deci, 2017).

In contrast to theories that explicitly focus on different qualities of motivation (e.g., AGT, SDT, and implicit theories), theories that simply differentiate types of motivation revealed less consistent findings related to cortisol. For example, nPower exacerbated cortisol response in one psychosocial-stress condition (Wegner et al., 2015); however, a lower cortisol response was observed in a nPower-inducing TSST relative to a friendly TSST (Wiemers et al., 2015). In win/loss contests, nPower was associated with an increase in cortisol in male participants after losing a contest (Oxford et al., 2017; Wirth et al., 2006); however, these findings were generally identified in a post hoc exploratory analysis. The link between nAffiliation and cortisol was also equivocal, with positive (Wirth & Schultheiss, 2006), negative (Wegner et al., 2014), and nonsignificant (McClelland et al., 1987) associations reported. nAchievement provided a more consistent relationship, predicting a dampened cortisol response during a visuomotor competition, during the TSST, and in response to negative feedback (Schultheiss et al., 2014, Studies 1 and 2; Yang et al., 2015). However, there were also experimental studies in which this relationship was not observed (Wiemers et al., 2015; Wirth & Schultheiss, 2006). Finally, studies grounded in reversal theory (Apter, 2001) did not evidence their hypotheses. Metamotivational states did not demonstrate any consistent relationship with cortisol (Cuevas et al., 2014; Filaire et al., 2007; Thatcher et al., 2003).

In summary, in a variety of situations, higher quality motivation was consistently associated with an attenuated cortisol response, and lower quality motivation was associated with an increased cortisol response, thus demonstrating a concomitant relationship (see Cacioppo & Tassinary, 1990). This finding has two potential implications. On the one hand, low-quality motivation can be viewed as provoking an adaptive physiological response aimed at mobilizing resources to help the individual manage the stress of social evaluation (e.g., Carver & Vargas, 2011). This conclusion, however, is overly simplistic. Despite the short-term benefits bestowed by cortisol release, acute cortisol responses are implicated with the suppression of acquired immune functioning (Segerstrom & Miller, 2004). Furthermore, frequent overactivation of the HPA axis (i.e., allostatic load; McEwen, 1998) is implicated with an increased risk for disease and dysregulated mental health (Lupien et al., 2009; McEwen & Stellar, 1993). Higher-quality motivation typically deemphasizes normative evaluation in favor of self-referenced improvement (Murayama, Elliot, & Friedman, 2012). Therefore, the more likely implication of the current review is that high-quality motivation may alleviate the perception of threat often associated with social evaluation, thus lowering physiological stress-related responses and facilitating long-term optimal functioning. The association between high- versus low-quality motivation and cortisol response may have implications for performance on complex and simple tasks. It is conceivable that low-quality motivation is better for simple performance requirements because the heightened cortisol response does not impede, or even facilitates, basic (e.g., repetitive) performance. On the other hand, the attenuated cortisol response associated with high-quality motivation is required for successful performance and engagement in complex tasks (see Cerasoli et al., 2014). Investigating these motivational processes would add a new dimension to the study of motivation.

Six studies, five based on MDT and one based on SDT, examined the relationship between motivation-related constructs and sIgA. The main finding in the MDT-based work was that nAffiliation, relative to nPower, was longitudinally and experimentally associated with an enhanced sIgA response. This association was found during times of acute stress (i.e., exam periods; McClelland et al., 1985), in response to need-provoking films (McClelland & Kirshnit, 1988), and over a 10-month academic calendar (Jemmott et al., 1983). sIgA represents a complex indicator of immune functioning but is identified as a marker of adaptive immune functioning (Bosch, Ring, de Geus, Veerman, & Amerongen, 2002). Nonetheless, immunoglobulins combat bacteria and viruses, trigger immune processes to target infection (Moser & Leo, 2010), and are therefore an indicator of heightened acute immunological functioning (Brandtzaeg, 2003). Thus, nAffiliation, relative to nPower, seems to be associated with immunoenhancing effects. Indeed, nPower is associated with greater self-reported illness (McClelland et al., 1980). Although MDT does not emphasize the quality of motivation as much as other theories considered in this review, these series of results imply a qualitative distinction between nAffiliation and nPower, at least regarding the modulation of immune function. The adaptive processes evident in this situation depends on the contrasting dominance of two needs and are also situation-dependent. This supports the key tenet of MDT that need strength can vary between individuals and contexts.

Assuming that nAffiliation leads to some degree of satisfaction of that need, this trend aligns with the large volume of literature documenting the physiological health benefits of social-support networks (for reviews, see Kemeny, 2009; Taylor, 2007). Indeed, the positive association of nAffiliation with a marker of adaptive immune functioning has important theoretical implications. Individuals with a higher degree of motivation toward affiliation with others would benefit most from the upregulation of adaptive immune functioning to combat viral infections commonly acquired via social contact. Furthermore, it has previously been demonstrated that perceptions of social connectedness may be more important than objective social contact in activating physiological responses, and this process may begin at the genetic level (Cole et al., 2007). The findings of this review further support the importance of psychological processes in modulating adaptive physiological responses for social contact and relatedness.

The SDT-based study demonstrated that thwarting of psychological needs was associated with an enhanced immune response (i.e., higher sIgA; Bartholomew, Ntoumanis, Ryan, Bosch, & Thøgersen-Ntoumani, 2011). At face value, this seemingly contradicts a key tenet of SDT because of a negative psychological characteristic associated with the upregulation of a marker associated with enhanced well-being. However, the Bartholomew et al. (2011) study was cross-sectional and, along with several MDT-based cross-sectional studies, may reflect a broad methodological weakness rather than a theoretical nuance. Endocrine-related responses are dynamic in nature and are sensitive to moment-to-moment variation. For example, cortisol is characterized by diurnal variation and sensitivity to caffeine, smoking, pregnancy, illness, and medication (Kirschbaum & Hellhammer, 1994; Smyth et al., 2013). Acute negative events are typified by an increased endocrine-related response, whereas chronic negative events are often, but not always, characterized by a flattened response (e.g., Miller, Chen, & Zhou, 2007; Taylor, Turner, Gleeson, & Hough, 2015). Many other hormones are sensitive to variation, for example, progesterone and estradiol during the menstrual cycle (Tortora & Derrickson, 2016). Furthermore, sIgA is modulated differently by acute- and chronic-stress reactivity (Bosch et al., 2002); however, this is not true for all markers of immune functioning (Segerstrom & Miller, 2004). In conclusion, measuring endocrine-related responses at a single time point is fraught with physiological and methodological pitfalls and should be avoided if possible.

A further theme that emerged from the MDT-based work is that winning a contest generally provoked increases in both testosterone and estradiol in participants high in nPower (Oxford et al., 2017; Schultheiss et al., 1999; Stanton & Schultheiss, 2007). However, this response did not occur to the same extent among winners who exhibited the more socialized form of nPower, in which power is associated with prosocial behavior (Schultheiss et al., 1999). In losing situations, there was often a nonsignificant relationship between nPower and testosterone. In other words, winning was positively associated with dominance-related hormonal responses in individuals who have a strong desire to demonstrate dominance. Testosterone is frequently associated with dominant behavior, as is estradiol in women to a lesser extent (Stanton & Schultheiss, 2007). For individuals who thrive on situations in which they can display dominance, winning, as opposed to losing, a contest bestows psychological and physiological benefits that prepare them for future dominance-related opportunities (Mazur & Booth, 1998). Thus, the associations between nPower and testosterone in competitive situations map well onto existing theories of dominance behavior, implicating nPower as a potentially important motivational process in this relationship.

There was also some evidence of a relationship between MDT theoretical constructs and progesterone. There was a significant, positive association observed between nAffiliation and progesterone (Wirth & Schultheiss, 2006), with evidence that this relationship may be moderated by individual versus team competition (Oxford et al., 2017). However, there were also nonsignificant associations between nAffiliation and nPower (Schultheiss et al., 2004), and nPower and nAchievement (Wirth & Schultheiss, 2006), with progesterone. Of the remaining endocrine-related responses, there were mixed findings regarding nPower, epinephrine, and norepinephrine (McClelland et al., 1987, 1985) and one positive association between nPower and sAA (Wiemers et al., 2015). Collectively, these findings do not allow for firm conclusions as to the relationship between MDT and these endocrine-related effects.

The analysis of the results of research into MDT also warrants scrutiny. Many of the studies did not support study hypotheses through parsimonious statistical analyses that directly matched study hypotheses. Instead, several studies relied on a post hoc analysis of single time points, separate analysis of subsamples, removal of participants, and flexible data-analytic strategies that were not adequately justified. In contrast, the AGT, SDT, and implicit-theory studies much simpler and conventional analyses that clearly matched study hypotheses. Furthermore, in instances in which a post hoc analysis was used, this was more clearly demarcated (e.g., Calvete et al., 2019; Yeager et al., 2016, Study 2). Thus, the evidence pertaining to MDT is not as robust as other theories included in this review.

### Similarity and compatibility across motivational theories

There has been a recent scientific effort toward unifying theories of motivation (Dweck, 2017; Uusberg et al., 2019). This approach delineates motivational processes into basic human needs that drive and energize behavior and mental representations that guide these goal-oriented processes. In this review, basic human needs relate to the SDT constructs of competence, relatedness, and autonomy and MDT needs for nAffiliation, nAchievement, and nPower. The evidence pertaining to the relationship among basic needs and hormonal responses was either limited for SDT or less consistent for MDT. The most robust finding was the association between increased nPower and decreased sIgA. Basic needs as defined by SDT have a functional synergy and are typically operationalized as a singular construct. Thus, for studies using questionnaire measures of basic need satisfaction and thwarting (i.e., Bartholomew, Ntoumanis, Ryan, Bosch, & Thøgersen-Ntoumani, 2011; Quested et al., 2011), our review was unable to disentangle the effect of individual need satisfaction on endocrine-related responses. In contrast, MDT needs are assumed to exist independently, a function of the individual-differences model on which they are based (McClelland, 1987). Hence, the individual relationships between MDT-based need strengths and endocrine responses are simpler to evaluate. This implies that we know more about endocrine-related responses as a function of individual differences in need strength than we do about psychological need satisfaction. There is broad agreement that when basic needs are thwarted (Bartholomew, Ntoumanis, Ryan & Thøgersen-Ntoumani, 2011), or when there is a discrepancy between implicit and explicit needs (McClelland, Koestner, & Weinberger, 1989), this results in poorer well-being. However, there was limited evidence in this review to extend this proposition to endocrine-related functioning (Bartholomew, Ntoumanis, Ryan, Bosch, & Thøgersen-Ntoumani, 2011; Schultheiss et al., 2012).

A key tenet of MDT posits that there are cultural and individual differences in need strength that are learned in early childhood (Schultheiss, 2008). In contrast, SDT places a greater emphasis on need satisfaction regardless of cultural or developmental differences (Ryan & Deci, 2017). One study in the review provides initial hormone-based evidence suggesting that a multitheoretical approach may have worth. The study integrated need strength and need satisfaction (Sieber et al., 2016) and demonstrated that individuals high in implicit need for autonomy experience lower sAA levels when their environment satisfied this need. This finding amounts to a replication research in which subjective well-being was the outcome of interest (Schüler, Sheldon, Prentice, & Halusic, 2016) and when the satisfaction of the implicit dispositions for competence and relatedness was examined (e.g., Hofer & Busch, 2011). This suggests that a multitheoretical approach may enhance understanding of need strength, need satisfaction, and their relationship with hormone responses with implications for well-being.

In contrast to basic needs, the importance of adaptive mental representations that guide behavior appears to clearer. Mental representations include mastery versus performance goals (Nicholls, 1984), incremental versus entity mindsets (Dweck, 2016), and autonomous versus controlled regulation (Ryan & Deci, 2017). One of the main findings of this review is the consistent and robust association between adaptive mental representations that motivate behavior toward need fulfillment and physiological markers of health (i.e., cortisol, sAA). In other words, across several theories low-quality motivation was associated with an elevated endocrine-related response compared with high-quality motivation. Multitheoretical approaches are becoming commonplace. For example, a theoretical review of AGT highlighted the potential for integration with SDT (Vansteenkiste, Lens, Elliot, Soenens, & Mouratidis, 2014). Research has identified the importance of autonomous functioning in increasing incremental mindsets (Job, Sieber, Rothermund, & Nikitin, 2018; Sieber et al., 2019). The current review suggests that similar multitheoretical work can be undertaken to examine physiological responses to different mental representations and the environments that support them.

The stronger evidence of the research into SDT, AGT, and implicit theories may have further theoretical implications. Implicit and explicit motivational systems tend to operate independently and influence behavior in different ways (Schultheiss, Patalakh, Rawolle, Liening, & MacInnes, 2011). In the same vein, it is plausible that explicit motivation (note that incremental and entity implicit beliefs are typically measured explicitly despite their label) may have a stronger relationship with endocrine-related responses than implicit-motivation constructs. However, concerns about whether implicit measures can distinguish between the desire to have a need met, the importance attached to a need, or the historical presence of a need have been raised (Ryan, Soenens, & Vansteenkiste, 2019). As a counterpoint, a recent randomized control trial used an intervention that successfully decreased motivational incongruence (Roch, Rösch, & Schultheiss, 2017). This intervention suggests a greater methodological sensitivity when measuring implicit needs than Ryan and colleagues acknowledge. Addressing these conceptual and methodological questions represents a fruitful area for future multidimensional motivation research.

### Strength and quality of evidence

The risk of bias was relatively low in the studies included in the review, with a few exceptions. The studies that scored lower on the bias inventory tended to be older (e.g., Dabbs et al., 1990; McClelland et al., 1980, 1987) and is perhaps indicative of the evolving standards of reporting in contemporary research. There was evidence of a risk of bias in the blinding of experimenters to condition, statistical power, and the reporting of exact probability values. Regarding double blinding, researchers should always consider the feasibility of double-blind designs to attain the highest-quality research. At present, this methodological option is not commonplace in broader psychological research. In contrast, exact probability values and power analyses are increasingly a requirement of psychological research in general, particularly following the replication crisis in psychology. On a related note, the cortisol studies based on AGT, SDT, and implicit theory all reported large effect sizes for their experimental studies (Hogue et al., 2013, 2017; Reeve & Tseng, 2011; Yeager et al., 2016, Study 1); however, only two studies reported a power analysis, of which one was underpowered. We do not draw any conclusions about the possibility of publication bias toward positive results, but it remains a threat to the validity and reliability of the observed effect (Button et al., 2013).

Concerns have been raised about the methodological validity, inferences that are made, and cases of circular reasoning in motivational research examining physiological markers (Richter & Slade, 2017). The motivation-based experimental conditions reviewed here demonstrated content and face validity, with studies frequently using successful manipulation checks to demonstrate concurrent validity. Predictive validity was evident; for example, higher-quality motivation predicted an attenuated cortisol response when faced with a moderate stressor, and greater nPower predicted higher testosterone and lower sIgA in more specific contexts. Finally, convergent validity was also demonstrated, as the respective SDT, AGT, and implicit-theory constructs of autonomous functioning, task involvement, and incremental theory all produced a theoretically coherent cortisol response to evaluative learning situations. Within the experimental studies, there was little evidence of circular reasoning, either within or between studies. All experimental designs followed a clear methodological pathway that contained a measure of motivation or a motivational manipulation, with the endocrine-related response of interest measured at baseline and at least one further time point during the experimental procedure. We could determine no subsequent experimental attempts to use the endocrine-related response as a predictor of the experimental procedure or as a motivational measure or manipulation. In conclusion, although the experimental studies included in this review seem to free of the issues raised by Richter and Slade (2017), researchers should remain vigilant of their concerns.

### Future directions and limitations

As discussed previously, none of the studies in this review included measures of behavioral outcomes or performance as part of their experiments (the win/loss studies had a predesignated winner, so performance was not objectively comparable) despite several having an evaluative element in the experiment (e.g., juggling, problem solving). This systematic review suggests high-quality motivation is implicated with an attenuated stress response. Investigating whether the physiological effects are associated with, or independent of, performance measures represents a worthwhile scientific endeavor. Relatedly, recent attempts have been made to integrate several conceptually related motivation theories (Dweck, 2017; Vansteenkiste et al., 2014). Indeed, empirical studies are more frequently adopting integrated approaches to investigate motivation-related phenomena (Chen et al., 2019; Job et al., 2018). Future research should consider investigating this multitheoretical perspective to further understand the relationship between theoretically distinct motivational constructs. Exploring whether there is an additive effect when integrating high-quality motivational constructs and measuring physiological outcomes or whether there is a ceiling to downstream effects is one possible line of investigation.

There is evidence to suggest that nAffiliation is positively associated with an adaptive immune response. However, why the desire for affiliation and related constructs, such as feelings of relatedness and social support, have such an effect of health and well-being remains unresolved (Taylor, 2007). Psychosocial states may trigger protective biological processes because of the enhanced risk of virus in social groups relative to more isolated states (Cole et al., 2007). The motivational and physiological processes implicated in these relationships warrant further investigation. For example, is the relationship between nAffiliation and sIgA mediated by fulfillment of the need, or is there a direct effect of nAffiliation on sIgA independent of social contentment? In other words, does the desire for affiliation trigger a proactive biological response in anticipation of fulfilling that desire (direct effect), or is the biological response a reaction to satisfying the need for affiliation (indirect effect)? This line of research has important implications for motivational science and for broader social support theories and their associations with physical and mental health.

Finally, this work was limited to published studies, dissertations, and theses only. In addition, non-English language studies were not considered. Although this is an acceptable method for systematic review, it may represent an incomplete picture of the literature. Despite adherence to methodological guidelines, subjectivity always remains a threat to the validity of a systematic review (Eysenck, 1994). This threat was mitigated by using two reviewers to screen the studies down to the inclusion stage, and I. M. Taylor also extracted a random sample of the included studies to check adherence to extraction protocols. We also acknowledge the boundaries by which we defined the inclusion criteria of both motivational constructs and endocrine-related responses may be subject to interpretation. Nonetheless, although researchers may differ in their methodological approach to systematic review, it is unlikely the results will be affected by significant divergence (Nieminen, Nicklin, McClure, & Chakrabarti, 2011).

### Conclusion

This review has been compiled to provide a scientific assessment of the current state of the literature concerning multidimensional motivation and salivary endocrine-related responses. There is experimental evidence that motivational constructs emphasizing higher-quality motivation produce an adaptive cortisol response in evaluative tasks. The robustness of this conclusion is enhanced by either successful replication or evidence in similar contexts. There is also evidence that nPower and nAffiliation are associated with lower and higher levels of sIgA, respectively. Evidence also exists within contextual situations, such as individuals high in nPower displaying increased testosterone when winning a contest; however, the evidence was not conclusive. The evidence revealed by this systematic review was also mapped onto a unified theory of motivation (Dweck, 2017). This framework allowed the comparison of findings from related theories and constructs and revealed several areas of theoretical alignment and compatibility. These findings have the potential to help refine theoretical aspects of the unified model and provide a more comprehensive understanding of motivational processes. Overall, the growing body of research helps us understand physiological responses to psychological phenomena and in turn has important implications for improved human functioning and well-being.

## Supplemental Material

sj-pdf-1-pps-10.1177_1745691620958008 – Supplemental material for The Relationship Between Multidimensional Motivation and Endocrine-Related Responses: A Systematic Review Click here for additional data file. Supplemental material, sj-pdf-1-pps-10.1177_1745691620958008 for The Relationship Between Multidimensional Motivation and Endocrine-Related Responses: A Systematic Review by Richard P. Steel, Nicolette C. Bishop and Ian M. Taylor in Perspectives on Psychological Science

## Transparency

*Action Editor:* Laura A. King

*Editor:* Laura A. King

## Notes

1. A consideration of nonsalivary measures would have led to the inclusion of one cross-sectional study that evidenced no significant associations between the need for affiliation and cortisol, epinephrine, and norepinephrine in plasma (McClelland et al., 1993).

2. As a predominant theory of motivation that is used in a wide range of theoretical and applied research, self-efficacy (Bandura, 1977) was included in the initial database search items. However, after reviewing the theoretical and methodological applications of self-efficacy research at the full-text stage, the reviewers unanimously agreed that it did not qualify as a multidimensional motivational theory, and all studies relating to self-efficacy were excluded.
