# Supplementary material for: The Relationship Between Multidimensional Motivation and Endocrine-Related Responses: A Systematic Review
Source: Perspect Psychol Sci. 2021 Jan 29;16(3):614–38. doi: 10.1177/1745691620958008 (PMC8114335; doi:10.1177/1745691620958008)
Supplement: sj-pdf-1-pps-10.1177_1745691620958008 – Supplemental material for The Relationship Between Multidimensional Motivation and Endocrine-Related Responses: A Systematic Review [file sj-pdf-1-pps-10.1177_1745691620958008.pdf]

Supplemental Material

Search Terms

Search ((((((((((((((((*endocrin*[Title/Abstract]) OR cortisol[Title/Abstract]) OR testosterone[Title/Abstract]) OR immunoglobulin*[Title/Abstract]) OR "IgA"[Title/Abstract]) OR "IgG"[Title/Abstract]) OR "IgM"[Title/Abstract]) OR ((hormon*[Title/Abstract]) NOT ((therapy[Title/Abstract] OR treatment)[Title/Abstract])))) AND Motiv*[Title/Abstract])) OR ((((((((((*endocrin*[Title/Abstract]) OR cortisol[Title/Abstract]) OR testosterone[Title/Abstract]) OR immunoglobulin*[Title/Abstract]) OR "IgA"[Title/Abstract]) OR "IgG"[Title/Abstract]) OR "IgM"[Title/Abstract]) OR ((hormon*[Title/Abstract]) NOT ((therapy[Title/Abstract] OR treatment)[Title/Abstract])))) AND Achievement goal*[Title/Abstract])) OR ((((((((((*endocrin*[Title/Abstract]) OR cortisol[Title/Abstract]) OR testosterone[Title/Abstract]) OR immunoglobulin*[Title/Abstract]) OR "IgA"[Title/Abstract]) OR "IgG"[Title/Abstract]) OR "IgM"[Title/Abstract]) OR ((hormon*[Title/Abstract]) NOT ((therapy[Title/Abstract] OR treatment)[Title/Abstract])))) AND (((self-determination[Title/Abstract]) OR "SDT"[Title/Abstract]) OR (("basic need*"[Title/Abstract]) OR "basic psychological need*"[Title/Abstract])))) OR ((((((((((*endocrin*[Title/Abstract]) OR cortisol[Title/Abstract]) OR testosterone[Title/Abstract]) OR immunoglobulin*[Title/Abstract]) OR "IgA"[Title/Abstract]) OR "IgG"[Title/Abstract]) OR "IgM"[Title/Abstract]) OR ((hormon*[Title/Abstract]) NOT ((therapy[Title/Abstract] OR treatment)[Title/Abstract])))) AND (((((entity belief*[Title/Abstract]) OR entity view[Title/Abstract]) OR entity theor*[Title/Abstract])) OR ((((Incremental belief*[Title/Abstract]) OR Incremental view[Title/Abstract]) OR Incremental theor*[Title/Abstract]))))) OR ((((((((((*endocrin*[Title/Abstract]) OR cortisol[Title/Abstract]) OR testosterone[Title/Abstract]) OR immunoglobulin*[Title/Abstract]) OR

"IgA"[Title/Abstract]) OR "IgG"[Title/Abstract]) OR "IgM"[Title/Abstract]) OR ((hormon*[Title/Abstract]) NOT ((therapy[Title/Abstract] OR treatment)[Title/Abstract])))) AND self-efficacy[Title/Abstract])) OR (((((((((*endocrin*[Title/Abstract]) OR cortisol[Title/Abstract]) OR testosterone[Title/Abstract]) OR immunoglobulin*[Title/Abstract]) OR "IgA"[Title/Abstract]) OR "IgG"[Title/Abstract]) OR "IgM"[Title/Abstract]) OR ((hormon*[Title/Abstract]) NOT ((therapy[Title/Abstract] OR treatment)[Title/Abstract])))) AND ((implicit power[Title/Abstract]) OR ((n power[Title/Abstract]) OR npower[Title/Abstract]))))) NOT ((Animal[Title/Abstract] OR mammal[Title/Abstract] OR "rat"[Title/Abstract] OR "rats"[Title/Abstract] OR mice[Title/Abstract] OR fish[Title/Abstract] OR monkey[Title/Abstract] OR chicken[Title/Abstract] OR flower[Title/Abstract] OR worm[Title/Abstract]))

Risk of Bias

Item	Description	Frequency x/41
1.	Is the hypothesis/aim/objective of the study clearly described?	38
2.	Are the main outcomes to be measured clearly described in the Introduction or Methods section?	41
3.	Are the characteristics of the participants included in the study clearly described?	32
4.*	Are the interventions of interest clearly described?	24/26
5.	Are the main findings of the study clearly described?	35
6.	Does the study provide estimates of the random variability in the data for the main outcomes?	36
7.	Have actual probability values been reported for the main outcomes except where the probability value is less than 0.001?	24
8. *	Was an attempt made to blind study subjects to the intervention they have received?	24/26
9. *	Was an attempt made to blind those measuring the main outcomes of the intervention?	4/26
10.	If any of the results of the study were based on data dredging, was this made clear?	38
11.	Were the statistical tests used to assess the main outcomes appropriate?	38
12.	Were the main outcome measures used accurate (valid and reliable)?	39
13.**	Did the study report the sample size required to detect a significant effect where the probability value for a difference being due to chance is less than 5%?	5

* Items 4, 8 and 9 relate only to the 26 experimental studies.

** The wording of this item was amended slightly to asses reporting of power analysis, rather than whether the study had sufficient power, with studies scored yes (1) and no (0).
